# Can online support groups address psychological morbidity of cancer patients? An artificial intelligence based investigation of prostate cancer trajectories

**DOI:** 10.1371/journal.pone.0229361

**Published:** 2020-03-04

**Authors:** Achini Adikari, Daswin de Silva, Weranja K. B. Ranasinghe, Tharindu Bandaragoda, Oshadi Alahakoon, Raj Persad, Nathan Lawrentschuk, Damminda Alahakoon, Damien Bolton

**Affiliations:** 1 Research Centre for Data Analytics and Cognition, La Trobe University, Bundoora, Victoria, Australia; 2 MD Anderson Cancer Center, University of Texas, Houston, Texas; 3 College of Engineering and Science, Victoria University, Heidelberg, Victoria, Australia; 4 NHS Trust, North Bristol, England, United Kingdom; 5 Department of Surgery, University of Melbourne and Olivia Newton-John Cancer Centre, Austin Hospital, Melbourne, Australia; 6 EJ Whitten Prostate Cancer Research Centre at Epworth Healthcare, Melbourne, Australia; 7 Division of Cancer Surgery, Peter MacCallum Cancer Centre, Melbourne, Australia; The Cancer Institute of New Jersey, Robert Wood Johnson Medical School, UNITED STATES

## Abstract

**Background:**

Online Cancer Support Groups (OCSG) are becoming an increasingly vital source of information, experiences and empowerment for patients with cancer. Despite significant contributions to physical, psychological and emotional wellbeing of patients, OCSG are yet to be formally recognised and used in multidisciplinary cancer support programs. This study highlights the opportunity of using Artificial Intelligence (AI) in OCSG to address psychological morbidity, with supporting empirical evidence from prostate cancer (PCa) patients.

**Methods:**

A validated framework of AI techniques and Natural Language Processing (NLP) methods, was used to investigate PCa patient activities based on conversations in ten international OCSG (18,496 patients- 277,805 conversations). The specific focus was on activities that indicate psychological morbidity; the reasons for joining OCSG, deep emotions and the variation from joining through to milestones in the cancer trajectory. Comparative analyses were conducted using t-tests, One-way ANOVA and Tukey-Kramer post-hoc analysis.

**Findings:**

PCa patients joined OCSG at four key phases of psychological distress; diagnosis, treatment, side-effects, and recurrence, the majority group was ‘treatment’ (61.72%). The four groups varied in expression of the intense emotional burden of cancer. The ‘side-effects’ group expressed increased negative emotions during the first month compared to other groups (p<0.01). A comparison of pre-treatment vs post-treatment emotions showed that joining pre-treatment had significantly lower negative emotions after 12-months compared to post-treatment (p<0.05). Long-term deep emotion analysis reveals that all groups except ‘recurrence’ improved in emotional wellbeing.

**Conclusion:**

This is the first empirical study of psychological morbidity and deep emotions expressed by men with a new diagnosis of cancer, using AI. PCa patients joining pre-treatment had improved emotions, and long-term participation in OCSG led to an increase in emotional wellbeing, indicating a decrease in psychological distress. It is opportune to further investigate AI in OCSG for early psychological intervention as an adjunct to conventional intervention programs.

## 1. Introduction

The difficult experience of dealing with cancer is unique to each patient. Based on individual needs, patients seek psychological and emotional support at different phases of cancer survivorship [1]. A recent study on psychological morbidity of prostate cancer (PCa) patients reports that most patients undergo increased emotional distress during the first few months of diagnosis, and highly likely to continue to suffer from emotional and psychological distress due to side-effects and negative impacts on Quality of Life (QoL) [2]. Another study highlights the prevalence of depression and anxiety in men with PCa across the treatment spectrum [3]. Positive outcomes of online support group participation have been highlighted in clinical trials [4,5], and these studies conclude that further investigations should be carried out to assess the long-term emotional benefit of OSG. However, due to the lack of data relating to emotional and psychological distress, most clinical decisions have been based on isolated research trials which are impacted by restricted sample sizes, treatment protocols and treatment stages [3]. This consequently increases the psychological morbidity experienced by patients during the entire cancer trajectory and may result in being left untreated. On the other hand, it has been reported that cancer patients benefit from frequent intervention therapies and that interventions in early stages of the trajectory assist in reducing psychological distress [6].

Cancer support groups were initially formed with the intention of addressing patients’ unmet psychological and emotional needs and providing peer-support where patients can exchange clinical information, share experiences and express emotions for the benefit of each other [7,8]. However, following the advent and prevalence of social media, support groups have been transformed into a new paradigm of Online Cancer Support Groups (OCSG) [9], creating global platforms that connect a multitude of cancer patients, across geographical and demographical boundaries[10] to provide a convenient, inexpensive yet effective resource for obtaining peer support [11]. Given the anonymity and wealth of information available in OCSG, recent literature indicates an increase in the use of OCSG by men with prostate cancer and reports that although men are reluctant to share their experiences in face-to-face conversations, they prefer the comparative anonymity of online communities [12,13]. Frequent communication with clinicians is limited [14], and men prefer to seek information and support via OCSG [15].

The advantages of OCSG have been acknowledged in randomised studies [16,17], but the potential for psychological intervention is yet to be examined. Given that online support groups are accessible and available 24 hours, 7 days a week globally, it has been observed that the constant support and sympathy received via online support groups can even surpass the patient expectations in a clinical setting [18]. In this study, authors investigate the use of Artificial Intelligence (AI) in OCSG to address psychological morbidity, with supporting empirical evidence from PCa patients. We extended a validated Artificial Intelligence framework, the Patient-Reported Information Multidimensional Exploration (PRIME) framework (CITE), to extract and analyse activities that indicate psychological morbidity; specifically, the reasons for joining OCSG, emotions expressed by patients, and the variation of deep emotions from diagnosis through to recovery. PRIME automatically extracted 277,805 conversations by 18,496 PCa patients in ten globally available, high volume online OCSG.

## 2. Material and methods

### 2.1 Participants

OCSG are organized as discussions initiated by a question, a comment or an experience that receives responses from other patients. An active, globally available, high volume OCSG is defined as having at least 100 new conversations per week [19]. The following table shows the OCSG selected for this study (Table 1).

**Table 1:** The patient distribution across the ten selected OCSG.

Online support groups	URL	n (% in total)
Healingwell	www.healingwell.com/community	2 520 (39.0)
Cancer orums	www.cancerforums.net	873 (13.5)
Cancer Survivors Network	csn.cancer.org/forum	810 (12.5)
Healthboards	www.healthboards.com/boards	429 (6.6)
Prostate cancer info link	prostatecancerinfolink.ning.com/forum	396 (6.1)
Cancer compass	www.cancercompass.com	356 (5.5)
Prostate cancer UK	community.prostatecanceruk.org	308 (4.8)
Patient info	patient.info/forums	299 (4.6)
Us too	www.inspire.com/groups/us-too-prostate-cancer	236 (3.7)
Macmillan UK	community.macmillan.org.uk	230 (3.6)

PRIME extracted data from these OCSG which yielded a dataset containing 609,960 conversations from 22,233 participants. This study only focused on patients with prostate cancer and excluded partners and/or caregivers, resulting in a cohort of 18,496 PCa patients (277,805 total posts). The study was approved by the human research ethics committee of La Trobe University (ID E16-145). All patient-reported data used in this study are non-identifying and publicly available from the corresponding OSG. The OSG are anonymized by design and we have only published aggregates of the analyzed data, which cannot be reverse engineered for any form of re-identification.

### 2.2 Study design

We used PRIME [20–22] to automatically detect relevant information from the posts, specifically emotions expressed and mentions of side-effects. This enabled creating an emotion and side-effect profile for each patient participating in OCSG, thus transforming their free-flowing, unstructured posts into ‘real-life’ patient-reported outcomes. PRIME has been successfully validated and demonstrated across several research endeavors; the extraction and investigation of patient emotions and clinical factors [23,24], the comparative analysis of patient-reported outcomes for different treatment types [25], and the study of online social influences on patient behaviours, decisions and emotions from diagnosis to recovery [19]. Outcomes of PRIME were compared with outcomes of three clinical trials [26–28] to assess the reliability and the validity of using PRIME [23] (Detailed information regarding PRIME is provided in S1 Fig).

To analyze the reasons to join OSCGs, we used Natural Language Processing [29] (NLP) and machine-learning based automatic topic extraction techniques to extract topics and sub-topics from the joining (initial) post of each patient. The title and content of each post were separately analyzed using an unsupervised machine learning algorithm, the Growing Self-Organizing Map (GSOM) algorithm [30] and the Latent Dirichlet Allocation algorithm [31], respectively. Each identified topic was further expanded into sub-topics using a semi-supervised keyword and key phrase extraction algorithm based on NLP techniques [32]. We have expanded this customised topic modelling technique as an extension to PRIME in order to capture the contextual information in OCSG.

**Fig 1:**
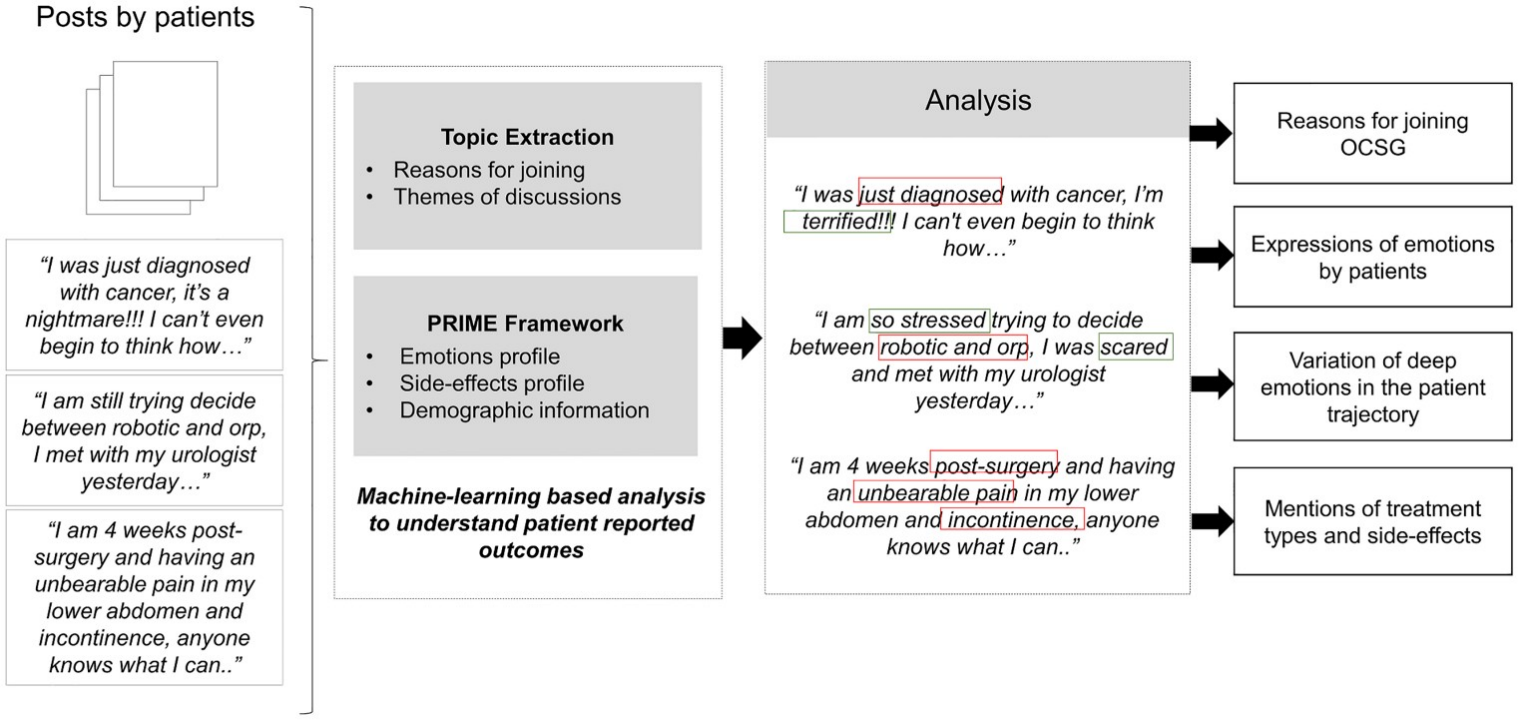
The extraction of patient-reported reasons for joining and emotions from a sample of anonymised OCSG discussions.

Subsequently, the PRIME framework was used to extract and analyze clinical and demographic information, side-effects profiles and emotions expressed by patients over 12 months. The extracted information was synthesized using the topics and sub-topics extracted prior. The following figure demonstrates a sample of anonymised first posts by patients, and how the machine-learning framework and NLP techniques were used to capture relevant information from unstructured patient conversations (Fig 1). In this study, we focus on three activities that indicate psychological morbidity, 1) the reasons for joining OCSG, 2) emotions expressed by patients, and 3) the variation of deep emotions from diagnosis through to recovery.

### 2.3 Statistical analysis

For the identified groups, we assessed the differences in the average number of emotion expressions over time. One-way ANOVA test was conducted to determine if there is a significant difference between the emotional expressions of identified groups. In scenarios with significant differences, Tukey-Kramer post hoc analysis was carried out to determine the significant group. Bonferroni correction was used as the correction mechanism (p<0.01; Bonferroni corrected). For the comparison between pre-treatment and post-treatment groups, t-tests were performed (p<0.05). To analyse if the emotional benefit outcomes were mediated by age, side-effects and Gleason score the characteristics of the two groups were analysed for the baseline characteristics.

## 3. Results

The following table shows the characteristics of 18 496 PCa patients selected for this study (Table 2). The characteristics were also automatically extracted using the NLP capabilities of PRIME.

**Table 2:** Patient characteristics (percentages calculated based on ‘Gleason score known’ population and ‘Age known’ population respectively).

	n (%)
**Total patients**	**18496**
**Gleason score known**	**7803 (42.19)**
< = 6	3895 (49.92)
7	2401 (30.77)
>7	1507 (19.31)
**Gleason score unknown**	**10693 (57.81)**
**Age known**	**10263 (55.49)**
<40	532 (5.18)
41–50	1331 (12.97)
51–60	2630 (25.63)
61–70	4237 (41.28)
>70	1533 (14.94)
**Age unknown**	**8233 (44.51)**
**Treatment type known**	**11816 (63.88)**
Surgery	7394 (62.58)
Radiation	3810 (32.24)
Active Surveillance	612 (5.18)
**Treatment type unknown**	**6680 (36.12)**

### 3.1 Reasons for joining OCSG

The automated intelligent analysis conducted using the previously mentioned topic extraction approach identified four distinct reasons for a patient to join the OCSG seeking information and/or emotional support. They are; (1) diagnosis, (2) treatment, (3) side-effects and (4) cancer recurrence. A majority of patients joined OCSG to seek information on treatment (61.72%), followed by diagnosis (17.40%) and side effects (9.12%), while far less for cancer recurrence (2.28%). Patients were then classified into four groups based on their intention to join OCSG (‘*diagnosis group’*, *‘treatment group’*, *‘side-effects group’*, *‘recurrence group’*). A further 9.48% of these conversations were general topics sharing other information. The sub-topic analysis (Table 3) revealed details on what had been most discussed in the identified groups.

**Table 3:** Main discussion topics and sub-topics discussed in the identified groups.

Topic category	Volume (%)
**Treatment**	**61.72%**
• Surgery options	18.93%
• Radiation	11.42%
• Active surveillance	5.86%
• Hormone therapy	8.80%
• Making decisions	5.45%
**Diagnosis**	**17.40%**
• Discussing test results	10.22%
• Asking for clinician recommendations	6.44%
• Seeking advice/experiences	6.60%
• Treatment options	0.85%
**Side-effects**	**9.12%**
• Urinary Incontinence	8.41%
• Erectile dysfunction	5.49%
• Other (Hernia, Tiredness, Stroke, Clot)	2.84%
• Bleeding and Infection	3.19%
**Recurrence**	**2.28%**
• Biopsy results	1.10%
• Metastasis cancer	0.93%
• Treatment/ therapy options	0.97%
• Active surveillance	0.35%
**General conversations**	**9.48%**
• Discussing PCa prevention	3.23%
• Experience sharing related to coping	0.88%
• Information on hospitals/new drugs/ Insurance	2.64%
• Diet/ Lifestyle	2.73%

### 3.2 The continuum of emotions expressed in the initial post

**Fig 2:**
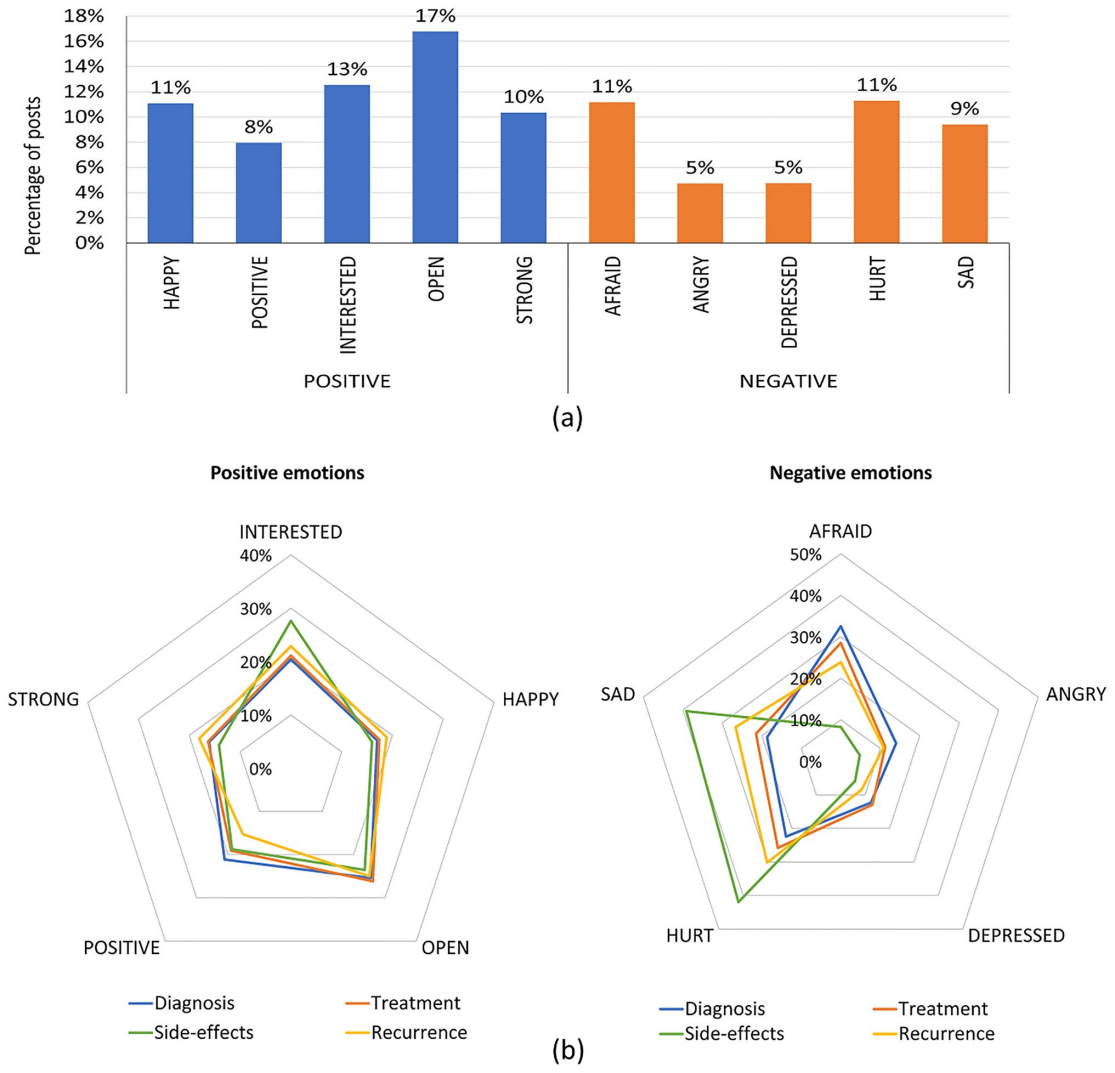
Continuum of emotions expressed in the first post by all participants (a) and the comparison of emotions based on the first post based on each identified group (b).

The spectrum of emotions expressed in the first post of each patient is depicted in Fig 2(a). The most frequently expressed emotions were ‘OPEN’, ‘INTERESTED’, ‘AFRAID’ and ‘HURT’. While the positive emotional expressions of feeling OPEN and POSITIVE denote the likelihood of patients to share their experiences and keen interest to seek information, expressions of being AFRAID and HURT indicate the anxious state of mind of patients when joining the OCSG. We further analysed the emotions across the identified four groups to determine associations between emotion expressed and the stage patients joined OCSG. The most frequent emotions across the four groups are shown in Fig 2(b), in which the scale denotes the percentage of posts for each emotion within the group.

The comparison between the four categories showed that positive emotion ‘INTERESTED’ was prominent in the side-effects group while ‘POSITIVE’ was slightly higher in the diagnosis group. In negative emotions, all groups except side-effects group showed increased expressions of ‘AFRAID’ which was higher in the diagnosis group.

As a noteworthy insight, the side-effects group showed a deviated pattern of negative emotions when compared to other groups. The side-effects group demonstrated increased emotional expressions related to the emotion ‘HURT’ and ‘SAD’.

This was further confirmed by a comparison analysis of aggregated positive, negative emotions among the four groups during the first month of joining the OCSG. It was disclosed that the side-effects group had exhibited increased negative emotions in the first month compared to the other three groups (p < 0.01). As demonstrated in Table 3 discussion topics, this is due to the psychological stress imposed by the physical pain and frustrations due to their side-effects.

### 3.3 Deep emotions in the cancer trajectory (1–12 months)

Using the PRIME framework, we were able to capture patient emotion fluctuations over a period of 12 months. The emotion intensity in each month was defined based on the average number of emotion expressions by each group. The analysis over a time period of 12 months demonstrated fewer negative emotions intensity at 12 months in all groups, where the diagnosis group showed the least negative emotions intensity at 12 months.

In order to determine the benefit of prolonged participation and long-term psychological effect, we then compared the average number of negative emotions exhibited in the 1^st^ month with emotions exhibited in the 12^th^ month across the four groups. In this comparison, all groups except the cancer recurrence group showed significantly less negative emotion intensity over time (p< 0.05). This is an indication that continuous support received from the OCSG and the benefit of joining OCSG at an early stage leads to a sustained emotional improvement.

### 3.4 Joining pre-treatment vs post-treatment: A comparison of emotions of patients with similar side-effects

In order to assess if there was a difference between the time of joining the OCSG, we compared two matched groups patients based on similar characteristics (age, Gleason score, and similar urinary and sexual side effects). Depending on the intention of joining, they were classified as patients who joined before treatments (pre-treatment group) and those who joined after treatments (post-treatment). The characteristics of the two groups with respect to age, Gleason score and side-effects are presented in Table 4.

**Table 4:** Characteristics of patient groups: Pre and post-treatment.

	Pre-treatment n (% in Pre-treatment)	Post-treatment n (% in Post-treatment)	n (% in Total)	*p* value	95% CI
**Total patients**	242	489	731		
***Gleason score***					
<7	148 (61.16)	288 (57.49)	436 (58.68)	0.3414	-3.9073 to 11.0285
7	65 (26.86)	165 (32.93)	230 (30.96)	0.0937	-1.0538 to 12.7669
>7	29 (11.98)	48 (9.58)	77 (10.36)	0.3148	-2.1475 to 7.6199
***Age groups***					
<40	7 (2.89)	10 (2)	17 (2.29)	0.4059	-1.2521 to 4.0991
41–50	36 (14.88)	70 (13.97)	106 (14.27)	0.7398	-4.2314 to 6.6497
51–60	98 (40.5)	189 (37.72)	287 (38.63)	0.4661	-4.6064 to 10.3048
61–70	79 (32.64)	163 (32.53)	242 (32.57)	0.9761	-6.9002 to 7.4131
>70	22 (9.09)	69 (13.77)	91 (12.25)	0.0684	-0.4111 to 9.1426
***Side-effects***					
Sexual symptoms reported	116 (47.93)	276 (55.09)	392 (52.76)	0.0671	-0.4930 to 14.7157
Urinary symptoms reported	191 (78.93)	420 (83.83)	611 (82.23)	0.1017	-0.9201 to 11.2096

**Fig 3:**
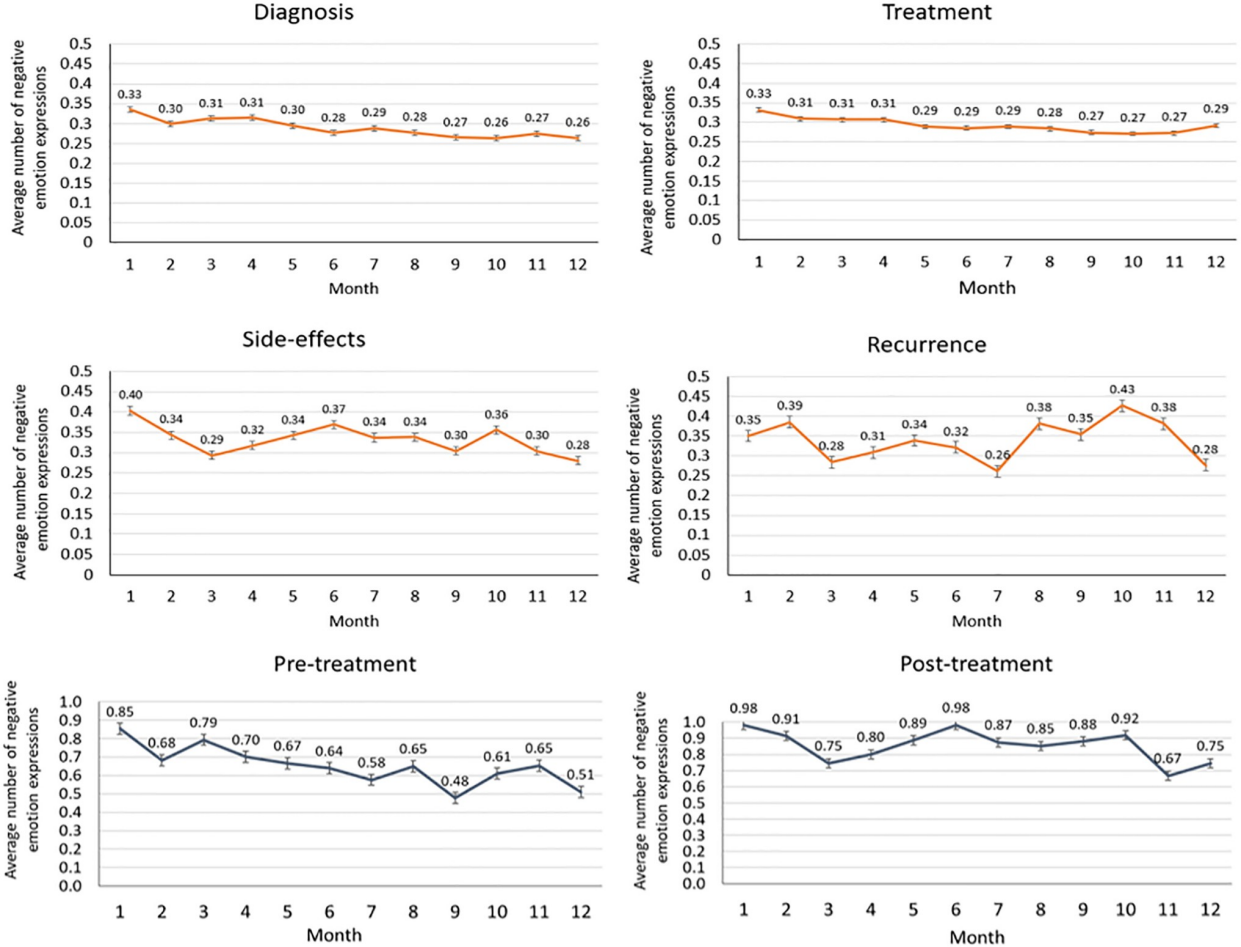
Negative emotions over a time period of 12 months analysed separately for the four groups; diagnosis, treatment, side-effects, recurrence and negative emotion fluctuations for pre-treatment and post-treatment groups separately (confidence intervals are based on standard error).

It was observed that patients with PCa who joined post-treatment had higher negative emotion intensity when joining the OCSG when compared to the pre-treatment group (p < 0.05), despite similar, age, Gleason score, sexual and urinary side effect profiles. Nevertheless, 12 months after joining the support group, both groups demonstrated improvement in emotions as the negative emotion intensity declined over time (p < 0.05; pre-treatment group mean change in negative emotions 0.424; post-treatment group mean change in negative emotions 0.4519) (Fig 3). The drop-out rate of patients in the 12-month period was approximately 8% per month. This was accounted for when calculating the aggregated negative and positive emotions in each month to offset the impact of participant drop-out.

However, it was observed that despite the improvement in both groups, at 12 months, the negative emotion intensity of the pre-treatment group was significantly lower than the post-treatment group (p < 0.05). We present this finding to confirm the benefit of joining OCSG at early stages of cancer, and the clear capability to position OCSG as a cost-effective and widely accessible early-psychological intervention program.

## 4. Discussion

The diagnosis of cancer induces psychological morbidity and a wide range of emotions, mostly fear, anxiety, and helplessness as patients transition from investigations to treatments with unanswered questions on pain and potential demise [33]. As a result, patients pursue informational and emotional support, and seek to be heard, reassured and supported by others[34]. Since each patient’s cancer journey is different and has individual needs and emotional support at different phases of the cancer survivorship [1], it is uncertain at which times PCa patients feel emotionally distressed and susceptible to experience depression and anxiety [3]. Using a validated artificial intelligence framework on large volumes of patient conversations in ten active global OCSG platforms, we provide insights on emotions of PCa patients across their cancer journey, which is otherwise limited by small sampled trials and treatment restrictions [3]. As OCSG are largely benefitting men with prostate cancer, OCSG provide a stable platform to analyse the psychological distress which can be often undiagnosed in clinical treatments.

Our findings highlight four distinct reasons for patients to join an OCSG; during diagnosis, treatment, side-effects and upon cancer recurrence. These four reasons for joining OCSG reflect four distinct transition periods which can be aligned with key decision points of the cancer journey of a patient [35]. Recent literature delineates that such transitions and decisive times cause patients to suffer from uncertainty and great emotional distress [33,36]. It has been further demonstrated that cancer diagnosis, treatment, coping with side-effects, after-care and recurrence have been identified as stages with high vulnerability for emotional distress [37]. Thus, using a detailed analysis from information on OCSG with a larger cohort of patients, our findings serve as empirical evidence that confirms the emotional and informational expectations of patients during cancer survivorship.

The results from the OCSG showed that most patients joined the support group seeking support for treatment (61.72%). This is also confirmed by thematic analysis on OCSG for PCa patients, which reveal that most patients discussed therapy recommendations [38] and searched for treatment information [39]. While existing work is limited to thematic analysis of PCa OCSG posts, we believe this is the first study to analyse patient emotional state when joining OCSG and fluctuations over time. Moreover, we found that patients who joined the OCSG seeking support on side-effects pursued increased emotional support as they expressed more negative emotions in their initial posts compared to other groups. The outcomes from the topic extraction convey that main discussions on their initial post were related to sexual dysfunctions and urinary incontinence. Encapsulating these results, we could infer that patients who join OCSG seeking advice and support on side-effects are more emotional compared to other groups. These findings are supported by current clinical studies which showcase the association between debilitating sexual and urinary side effects and depressive symptoms [40,41].

Based on our comparison study of emotion when joining and after 12 months of participation in OCSG, it was observed that negative emotion intensity of all groups decline over time, while this change was significantly visible in the diagnosis group. We postulate the decreased emotion intensity at 12 months is due to the continuous support and advice received from the OCSG [42]. The significant emotion improvement of the diagnosis group indicates the value of early participation in OCSG for long-lasting emotional support.

We further compared the emotions of patients (matched age and Gleason score) who had reported similar side-effects but had joined the OCSG at two different points; before and after treatment. Although both groups benefited from the prolonged participation in OCSG, the negative emotion intensity of patients who joined prior to treatment was significantly lower when compared to patients who joined after treatment. This serves as evidence to communicate the significant role of joining OCSG during the early stages of a patient journey. As the observations from psychological studies state that emotional support and psychological intervention should be targeted around the time of the initial diagnosis and treatment stages [43], joining OCSG at early stages will be more effective in assisting patients to cope with distress induced by cancer.

We position our findings as evidence of the significance of OCSG for early psychological intervention, adjunct to routine clinical care. This study is a first to demonstrate that joining an OCSG leads to similar benefits to those gained from conventional psychological interventions of cancer patients [6].

As clinical implications of this study, we recommended that clinicians inform newly diagnosed patients on the availability of OCSG as a conveniently accessible source of informational and emotional support; and the psychological benefits gained participating in OCSG conversations since early stages. Moreover, health services and cancer-care organisations are encouraged to initiate and resource OCSG platforms [18], in order to make it more accessible to patients. It has also been reported that the level of support a cancer patient receives from family and friends tends to decrease over time, and OCSG can be a form of long-term on-going peer support that fills this gap [44]. This can contribute towards improved QoL as patients will receive continuous care and emotional support which is deemed necessary throughout the cancer journey from diagnosis and treatment to after-care [45].

In terms of limitations, any study on computer-mediated communication is influenced by the inherent selection bias as the selected sample represents a group who are computer literate and are aware of such online groups. However, in order to minimize the selection bias of those who utilize OCSG or social media, we have previously validated the findings by comparing with large randomized controlled trials [25]. Thus, we assert that the outcomes of this study could be effectively utilized to suit a larger population by means of a prospective study. Future research directions in analyzing OCSG could leverage advances in artificial intelligence to automatically identify patients with great emotional distress, which can be directed for immediate clinician intervention, thus elevate the current patient care. Such early intervention would improve individualized care, enable better QoL among patients and would positively contribute to the current higher suicide rates of distressed patients. In conclusion, we highlight the importance of facilitating the setup of institution-based OCSG and encouraging the use of automated intelligent analysis of the large volume of data to provide patient-centred insights in order to assist clinicians further enhance individualized cancer care.

## 5. Conclusion

In summary, we have investigated the role of OCSG in addressing psychological morbidity of cancer patients using PRIME, a validated AI framework on a dataset of 277,805 conversations by 18,496 PCa patients from ten international OCSG. The results indicate PCa participation led to a decrease in psychological distress, where patients joining pre-treatment had improved emotions, and long-term participation in OCSG led to an increase in emotional wellbeing. Both results and implications of results confirm the practical validity and healthcare value of using AI in OCSG for early psychological intervention as an adjunct to formal intervention processes.

## Supporting information

S1 Fig Information regarding the PRIME framework (taken from the original publication). (DOCX)
