# Supplementary material for: Can online support groups address psychological morbidity of cancer patients? An artificial intelligence based investigation of prostate cancer trajectories
Source: PLoS One. 2020 Mar 4;15(3):e0229361. doi: 10.1371/journal.pone.0229361 (PMC7055800; doi:10.1371/journal.pone.0229361)
Supplement: S1 Fig — (DOCX) [file pone.0229361.s001.docx]

**Supplementary Material**

**Figure 1: Information regarding the PRIME framework (Taken from the original publication)**

**De Silva D, Ranasinghe W, Bandaragoda T, et al. Machine learning to support social media empowered patients in cancer care and cancer treatment decisions. PLOS ONE. September 2018.**

**
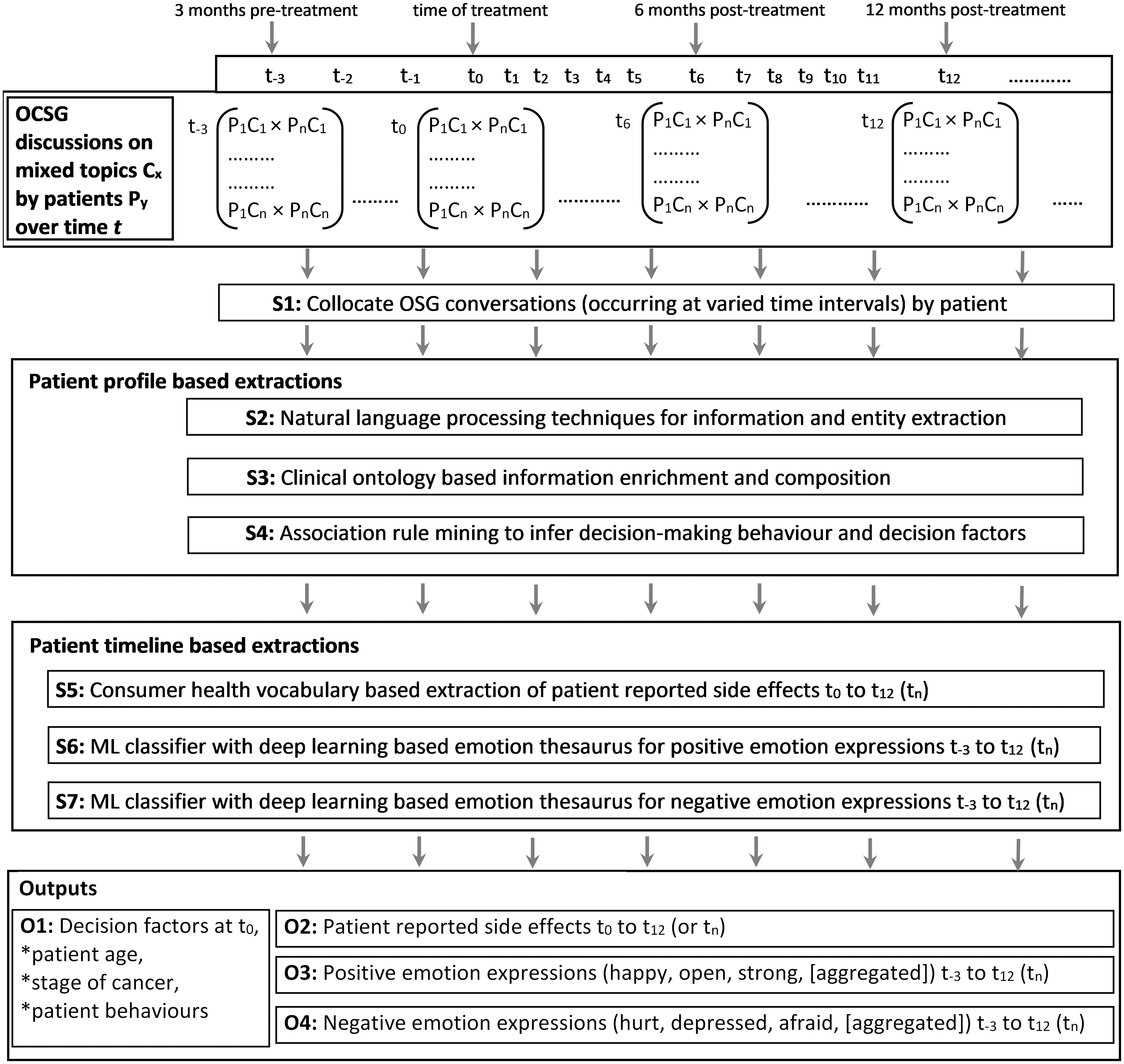
**

**Intelligent extraction of clinical factors**

An OSG comprises a large number of discussions where patients contribute their decisions, experiences and opinions at different stages of their patient journey from diagnosis to post-treatment. The naturally occurring order of discussions provides a multitude of granular and aggregate information on patient behaviours, side effects and emotion expressions over time. However, posts by a single patient are scattered over multiple discussions. Therefore, S1, collocates conversations by a single patient, chronologically ordered based on timestamp. In S2, NLP based information retrieval techniques are used to process the text corpus and subsequently, machine learning algorithms for classification are utilised to extract demographic information mentioned in free text Next, S3 enriches this multidimensional information model with prostate cancer specific clinical information, which are important to categorise patients based on the stage of cancer. In relations to prostate cancer, Gleason and PSA information are key determinants that are extracted. In S3, association rules and extracts from clinical ontologies are utilised to capture multiple narrative styles for Gleason and PSA mentions(e.g., ‘GS3+3’, ‘Gleason 7’). Subsequently, a classifier based on regular expressions was developed to capture the numerical details of Gleason and PSA scores.

**Intelligent extraction of side-effects over time**

Stage S5 onwards, PRIME framework incorporates the time dimension of OSG discussions and patient interactions. A patient event timeline is automatically generated for each individual based on the self-disclosed side effects captured in S5 and positive/negative emotions captured in S6-S7. Each patient timeline is time-normalised by considering the treatment month captured in S4 as t0. The events (side effects and emotions) are aggregated monthly based on the reported timestamp, and the timeline is generated from three months pre-treatment (t-3) to 12 months post-treatment (t12) based on the available information. S5 captures the self-disclosure of side effects and grouped into four key categories: urinary, sexual, bowel and otherwhich represent side effects of prostate cancer treatments. Note that, other represents the miscellaneous side effects such as hernia, clots etc. A thesaurus of relevant terms (words/phrases) was used to capture any mentioned of an occurrence of side effects and map such mentions to the timeline based on the associated timestamp. Even though the clinical terms for side effects are well defined and recorded in clinical ontologies, individuals often describe side effects using everyday language (e.g., urinary incontinence described as leakage, leak, drip), which are not found in clinical ontologies. Therefore, a sample of OSG posts was examined by a team of clinical experts, and consumer health terms related to each side effect category were captured and included in the thesaurus.

**Intelligent extraction of emotion over time**

As established in the clinical literature, OSG are an accommodative environment for patients to freely express emotions. Expressions of emotion reflect Quality of Life (QoL) measures such as living with the condition, the impact of treatment preferences and side effects. In S6, a machine learning technique incorporating a domain-specific vocabulary of positive emotion expressions determines explicit and implicit instances of positive emotions, emotion categories and associated strength of emotion and in S7, this was extended to negative emotion expressions.

Many psychological emotional models have been proposed in the research literature to represent human emotions. These range from the two-dimensional valence-arousal model to multi-dimensional models such as emotion wheel. While such models serve as the theoretical basis for emotion representation, computational implementations must capture expressions of emotion from textual discourse. For example, sentiment analysis techniques are the computational implementation of the valence-arousal model, which provide a signed real-value as the sentiment score, where the sign (positive/negative) represents the valence and the absolute value of score represents arousal. Although sentiment analysis techniques are relatively mature and commonly used for capturing emotions, the two dimensional model is coarse-grained for representing complex emotional states of OSG users. Therefore, we developed a new machine learning technique based on the Emotion Wheel to capture a multi-dimensional representation of emotions.

Emotion Wheel has eight primary emotions (joy, trust, surprise, sadness, disgust, anger, anticipation and fear) and further eight secondary emotions which are derived using combinations of primary emotions (e.g., love: joy+ trust). These 16 emotions (primary and secondary) specified in the Emotion Wheel were incorporated as the emotional dimensions in the proposed computational model. The emotional intensity of each emotion is determined based on the proportion of relevant emotional terms present in each OSG post, resulting in a 16-dimensional real-valued emotion vector for each OSG post.

Expanding a seed list of lexicons is a tedious activity, which is often achieved using crowdsourcing techniques such as Amazon Mechanical Turk. However, recent research reports a semi-supervised deep learning approach using word-embedding. Word-embedding learns dense vector representations of words and phrases while automatically preserving the semantic relationships that exist in the text corpus by incorporating such relations into the vector space of the word-embedding. This enables the use of linear algebra to capture different semantic relationships within word-vectors in the word-embedding. The famous example in shows that the vector arithmetic of word vectors ‘King -Man + Woman’ results a word vector similar to the word vector of ‘Queen’.

Developing such a word-embedding using OSG discussions enables to capture terms used by the OSG users that are semantically similar to the seed emotional terms. We have developed a word-embedding from a large text corpus which contained a total of 4,795,428 OSG posts. This corpus was pre-processed to remove URLs, convert to lower case and then separated into sentences using the Punkt sentence tokenizer available in python NLTK library, which has shown state-of-the-art performance when compared to other sentence tokenizers with over 90% accuracy on user generated content. This tokenization has resulted in 36,222,536 sentences. This text corpus was used to train a 200 dimensional word-embedding using Word2Vec technique with skip-gram model and negative-sampling. We utilised the python genism library for this implementation. The resulting word-embedding contains 312,196 unique terms (words and phrases).

Following the trained word-embedding, top 25 most similar terms for each seed term in the emotion thesaurus was identified using a nearest neighbour search in the embedding space using Cosine similarity. These identified terms are semantically similar terms to the seed emotion terms, in which some of the terms have the same emotional sense of the seed term while some others may not. For example, the top five nearest neighbours of sorrowful are sadness, sincerity, joyful, and deeply saddened, in which joyful is semantically similar but has the opposite emotional sense

Intensity modifier terms are a set of terms that increase or decrease the intensity of the emotional term. For example, the term ‘very’ increases the intensity of the emotion ‘good’ when used together, whereas, the term ‘kind of’ decreases the intensity of the emotion ‘okay’ when used together. Moreover, some terms completely negate the emotions e.g., ‘not okay’ negates the emotion expressed by ‘okay’. A thesaurus of such terms are often used in rule based sentiment analysis tools such as SentiStrength and VADER to improve the accuracy of the sentiment score. In this work, we have used the intensity modifier term thesaurus used in VADER.

In summary, as explicated above, PRIME functions in seven stages S1-S7 to transform OSG discussions from unstructured text discussions in the everyday language into multi-granular, multidimensional information individualised by the patient to analyse and aggregate ‘real life’ patient reported outcomes.
